# The Hospital. Nursing Mirror

**Published:** 1898-07-30

**Authors:** 


"The Hospital" Nursing Mirror.

Being the Nursing Section of "The Hospital."

[Contributions for this Section of "The Hospital" should be addressed to the Editor, The Hospital, 28 & 29, Southampton Street, Strand, London, W.C., and should have the word "Nursing" plainly written in left-hand top corner of the envelope.]

### News from the Nursing World.

### THE ROYAL RED CROSS IN BULUWAYO.

Just a month ago, on June 21st, the Acting Deputy-Administrator of Rhodesia, Central Africa, presented Mother Jacoba with the insignia of the order of the Royal Red Cross. This order, which was instituted by the Queen on St. George's Day, 1883, is awarded for zeal and devotion in providing for and nursing sick and wounded sailors, soldiers, and others in the field, on board ship, or in hospital. It is only given for conspicuous service, and there are, exclusive of Royal personages, only 68 members. Mother Jacoba, who receives her decoration for devotion to the sick and wounded in Rhodesian hospitals during the last seven years, especially during the late Matabele rebellion, was born at Wurtemberg, and is a nun of the order of St. Dominic. Roman Catholic nurses may be glad to know that the sisters will welcome thoroughly-trained recruits. A serious religious vocation and a certificate are the necessary qualifications, and we hear that the Rev. A. M. Daignault, 114, Mount Street, London, W., is able to furnish full particulars.

### NURSES AT WESTBROOK.

The nurses at the Meath Home of Comfort for Epileptic Women and Girls, Westbrook, Godalming, are fully trained ladies. They are under the rule of a lady superintendent and an assistant matron. Their bed-rooms are comfortable, and possess an advantage sometimes found in buildings adapted to and not erected for a public purpose, in that they are much larger than is usually the case. In addition, the nurses have a sitting-room away from the house where they can ride all the hobbies dear to women without let or hindrance. It is a "converted" out-building, and the walls have been panelled and varnished three parts up to the ceiling. It is furnished with a solid-looking round-table, easy chairs, and a sofa, which is a recent acquisition. The means of making a private cup of tea are handy, and bicycles are allowed to find a place in a corner. Nurse Punchard, the assistant matron, was the first nurse chosen when the home was founded, and is a tower of strength, especially with cases during a fit, and her committee in electing her to her present position expressed "their deep appreciation of her services to the Home." Miss Anderson, the first lady superintendent, was obliged to resign last year on account of ill health; her place has been taken by Mrs. Cousins. In due course it is desired to establish a cottage where nurses and patients may both be sent for a spell of rest and change. One at Ottershaw, lent by the Countess of Meath last September, proved of great benefit to those who were able to visit it.

### A CHILDREN'S TREAT.

The Earl and Countess of Jersey have again generously invited about 600 boys and girls of the National Refuges for Homeless and Destitute Children, including the sailor boys of the *Arethusa* and *Chichester* training ships, Greenhithe, Kent, and 200 girls from Sudbury and Ealing, to spend the day at their beautiful park at Osterley, Isleworth, on the 29th inst. The invitation has been gratefully accepted, and the brass bands of the Homes and training-ships will attend.

### THE "NEST," BANSTEAD.

A delightful afternoon was recently arranged for the crippled lads now inmates of the "Nest"—the surgical home for boys, at Banstead, Surrey—by the chairman of the local committee, the Hon. Mrs. Randall, assisted by Mrs. Fearon, Mrs. Acland, and Mrs. Garton. The patients were installed in flower-bedecked chairs in the garden, where Punch and Judy amused them excellently until the arrival of a gay procession of mail-carts, baby carriages, and toy wheelbarrows, with a pony, a donkey, and a bicycle. Prizes were afterwards given to the child whose decorations were the prettiest, and then followed tea—strawberries and cream—the gift of Mrs. Garton, and subsequently "Punch" gave another dramatic performance. The home is a small one, containing about twenty beds, and is usually full. Its doors are open to boys before, as well as after, surgical operations, a boon of considerable value.

### OUR CHRISTMAS GIFTS.

Christmas is a long way off, yet it is not so far away that we can forget to prepare for our annual distribution of clothing to the convalescent patients leaving the warm hospital to face the cold blasts of December. Everyone now is bent on holiday-making, and many are by no means sorry to have a piece of needlework on hand to keep their fingers usefully employed during the hours spent out of doors in country gardens or by the seaside. We are anxious this year to distribute complete suits of clothing, and have already promises of suitable garments. Warm vests, petticoats, stockings, shirts—of all kinds and sizes—mittens, comforters, anything and everything in the way of clothing that will protect delicate bodies from the cold. With this timely reminder, we will trust our readers to make "The Hospital Clothing Distribution" a generous one.

### THE GUILD OF ST. VERONICA.

The Bishop of Southwark invited the nurses belonging to the Guild of St. Veronica for Nurses to celebrate their thirteenth anniversary in the grounds of Denmark House, Lewisham Hill. Advantage was taken by nurses of the occasion to present the Superior of the Guild, the Rev. J. Dixon, with a token of their appreciation of his sympathy and counsel. Canon Jelf preached at the service subsequently held in Lewisham parish church. A fair number of members and their friends attended both the gathering and the service. The guild, of which the secretary is Miss Darnell, residing at The Sanatorium, Clifton, aims at meeting the special religious difficulties of nurses.

### PAYING PROBATIONERS.

The Birmingham Board of Guardians has, from time to time, reduced the number of probationers who pay for their training. The original number was forty; a year ago it was twenty; and, at a recent meeting, it was further reduced to ten. Each lady, under the old system, paid a fee of £20 for a year's training, and, upon receiving a certificate at the end of it, as a rule sought and found remunerative work elsewhere. However satisfactory this may be from the paying probationer's point of view, the Guardians have found that it is better worth their while to spend an additional £300 a year in order to pay their probationers £10 per annum, and to bind them to the service of the infirmary for a term of three years. Incompetent work is expensive, and too many inexperienced nurses cannot but be injurious to the interests of the patients, for if teachers be lacking the nurses must learn their work, if they learn it at all, by experiment. On the other hand, insufficient training is prejudicial to the future career of the nurse herself. She is disqualified for the better positions; and often she can only command lower rates of payment. If a three-year certificate be a *sine qua non* to the successful nurse, the action of the Birmingham Board of Guardians is, therefore, strongly to be commended from the probationer's point of view.

### THE NATIONAL UNION OF WOMEN WORKERS.

The National Union of Women Workers (Berners Street, W.) has issued a series of penny tracts, written especially for workers amongst the poor. District nurses will find them most useful, especially Nos. 2, 3, and 6. No. 1 is by Mary Clifford, guardian at Barton Regis, and treats of "Out Relief"; No. 2, "Hints for District Visitors on the Important Subject of Sanitation"; No. 3, "Legal Difficulties of the Poor," is by a barrister; No. 4, "Children's Country Holidays"; No. 5, "Girls' Clubs"; No. 6, "The Administration of Charitable Relief." Our readers may remember we gave a short notice of the National Woman's Union some time ago, the first conference of which was held last year at Croydon. This year's conference will be held in the Prince's Street Rooms, Norwich, in October, when papers upon the work of maternity nurses, the care and nursing of the epileptic, of deaf mutes, and of the feeble-minded will be read.

### NURSES FOR COLCHESTER.

An influential company assembled on the 18th inst. in the Town Hall, Colchester, to consider the founding of a nursing association for the town in affiliation with the Queen Victoria's Jubilee Institute for Nurses. Alderman James Paxman (the Mayor) presided, and said that the need of such an institution was greatly felt in the borough, and that everyone was aware that a large amount of suffering went on amongst the poorer classes, particularly when there was a slight epidemic in the town. Miss Amy Hughes, secretary of the Nurses' Co-operation (New Cavendish Street, London), gave a practical address on the importance of the poor enjoying the help of thoroughly competent nurses. Dr. Fenn next spoke of the general recognition by medical men of the importance of trained nursing. The Mayor was elected president of the new association, and Miss Benham and Miss Marriage hon. secretaries. Two nurses are to be engaged as a beginning, and it is hoped that in due course the work will develop into a centre for supplying nurses to the whole town.

### NOTABLE NURSES.

Sister Dora taught the Walsall people to love her, and those belonging to her calling. They reverence her memory, and her example inspires many disciples. All over the world "Walsall nurses" may be met; and a correspondent of one of the local journals gives a rough summary of a few of the most noted amongst them. It would be far from an unprofitable labour if each town would compile a roll-call of nurses trained in its schools, and keep some record of their achievements in after life. Such a record might be an inspiration to those entering upon their training, and help to bring into great prominence the holier aims of a noble calling.

### SHORT ITEMS.

In view of fulfilling the increasing demand for the services of nurses, it was decided at a general meeting of subscribers of the Hampshire Nurses' Institute, on the 7th instant, to employ, in addition to those resident in the home, other nurses on the co-operative system. This resolution in no way affects the original working of the institution, which is regarded as satisfactory.—A concert was given recently in aid of the Epsom District Nursing Association, by Mr. Clifford Essex and the Pierrot Banjo Team.—The Hoxne Board of Guardians, after a long discussion, voted a donation of two guineas to the Nursing Association at Worlingworth.—Miss Ellen Grant, for some time superintendent under the Holborn Union, has been appointed night-sister under the Hampstead Board of Guardians.—The Nursing Association of Thetford has just discontinued the services of the cottage nurse for the best of all reasons, namely, there is no work for her. The district nurse is still retained, and a special nurse will be engaged in case of necessity.—The demand for nurses at the Dorset County Home for Nurses, Dorchester, has been so great that it has been found necessary to furnish another bedroom.—Nurse Despard, of the Adelaide Hospital, Dublin, was thrown from a hackney car on the 19th inst., and seriously injured. She was taken to the Jervis Street Hospital, where she remained.—A series of three garden parties and sales of work have been given in aid of the district nursing branch of the Leicester Institution of Trained Nurses. The third was held in the grounds of Mr. T. Fielding Johnson at Brookfield, Stoney Gate. Lady Mary Carr-Glyn opened the sale, and two short dramatic performances were given by amateurs.—The managers of the Edinburgh Royal Infirmary recently approved the adoption of a report recommending the principle of instituting a home of rest for their nurses.—There has recently been formed in Sydney, New South Wales, a nurses' guild, which is allied with the Guild of St. Barnabas. On St. Barnabas' Day a special reunion was arranged, appropriate services being held in St. James's Church, and a social evening spent in the Parish Hall.

### Antiseptics for Nurses.

BY A MEDICAL WOMAN.

### XI.—CHINOSOL AND OTHER ANTISEPTICS.

DURING the last year a new antiseptic called "chinosol" has been introduced and has attracted some attention. It is derived from an oily liquid known as "quinoline," which occurs in coal-tar, and is described as having a pungent, aromatic, and very disagreeable odour, a bitter acrid taste; and as turning brown when it has been exposed to the atmosphere. Chinosol, however, itself has none of these disagreeable properties, but is a light yellow crystalline powder which dissolves very readily in water. Chinosol has also a slightly aromatic odour and an astringent taste. It possesses very marked antiseptic, disinfectant, and deodorant powers.

Chinosol can be obtained in two forms for surgical purposes, either as a solution or as a powder. For washing out wounds, cleansing the hands, and other similar uses, it can be prepared in solutions of different strengths varying from 1 part in 60 to 1 part in 1,000 of water, and when thus employed it causes no irritation to the skin. One great drawback to chinosol is its effect upon steel instruments, and if these have to be kept in this antiseptic for any length of time, a much weaker solution must be used, viz., one not stronger than 1 in 2,000, otherwise it will have disastrous effects on the steel. Chinosol destroys the edges of cutting instruments, and deposits greenish-black spots on all, which are very difficult to remove, and it also discolours and roughens white bone handles.

Chinosol may also be used as a powder when a dry antiseptic dressing is required. It should, however, then be mixed with boracic or starch powder, as when used pure chinosol is apt to cause painful irritation in a wound. It also discolours a raw surface, turning it a dark brown colour. None of these drawbacks, however, are connected with chinosol when it is used as a lotion, and, moreover, experiments have proved that it acts as a better antiseptic in solution than in powder.

Chinosol can be obtained in the form of tabloids, each containing 15 grains, with which solutions of any required strength can be quickly prepared by merely adding water. It can also be had as powder as in the following antiseptic dressings, viz.: Wool, lint, gauze, and tow, all of which are prepared in two strengths—a 1 per cent. and a 3 per cent. Chinosol has been proved to combine "large antiseptic properties with a relatively small power of doing harm."

Oxycyanide of mercury has come into use as an antiseptic more recently than perchloride of mercury. Both are equally poisonous, but the oxycyanide possesses one great advantage over the perchloride salt, in that it does not affect instruments injuriously, and has consequently a wider use. It is non-volatile and almost entirely non-irritating, and is used in solutions of the same strengths as the perchloride.

Dermatol is the name given to an antiseptic which has lately been introduced into surgical practice in Germany and France. It is a compound of bismuth and gallic acid, and hence is chemically a subgallate of bismuth. It is a yellow powder, which is insoluble in water and quite odourless. It has been favourably reported on as an antiseptic application for small external wounds and raw surfaces, but owing to the fact that the antiseptic properties of bismuth, which forms the main part of dermatol, are not great, it cannot rank amongst the first class antiseptics. Its action is in fact rather astringent than antiseptic. Dermatol can also be prepared as an ointment combined with vaseline.

Europhen: Fournier points out that the two great disadvantages of using iodoform as an external application to wounds are, first, its objectionable odour, which seems to pervade everything, and is nearly always strongly objectep to by the patient on the grounds that everything smells and tastes of iodoform; and second, that occasionally it has produced toxic effects, *i.e.*, the well-known symptoms of so-called "iodoform poisoning," with high temperature and loss of appetite. In europhen iodine is combined with a cresol compound, and when in the presence of an alkali it is said that iodine is freely produced, and that this change occurs whenever the powder comes into contact with an ulcerated surface. Consequently, europhen, like iodoform, with which it is chemically similar, owes all its antiseptic properties to the iodine it contains, and which can be liberated. Thus, europhen cannot be regarded as at all a powerful antiseptic, though doubtless very useful as a dusting powder for ulcers. It is insoluble in water, but is soluble in alcohol, ether, or chloroform, and is very readily soluble in fixed oils. It has only a very slight, hardly perceptible odour, which in fact is rather pleasant than otherwise. Other modifications of iodoform, intended to obtain the advantages of the iodine as an antiseptic, but to conceal the disagreeable odour, have been recommended by Berkeley Hill. These are iodo-carbon paste, which contains finely-powdered iodoform, 1 drachm; wood charcoal powdered, 2 drachms; glycerine of starch, 2 drachms; glycerine, 1 drachm; and oil of lavender, 20 minims; or else iodoform powder can be used dissolved in eucalyptus oil in the proportions of 1½ drachms iodoform powder in 1 ounce of eucalyptus oil and 5 ounces of olive oil. Other means of disguising the odour of iodoform are by adding coumarine, which is obtained from the Tonquin bean, in the proportion of 50 parts to each part iodoform, or else finely-ground coffee in the proportion of 1 to 3.

Iodol is a compound obtained from iodine, potash, and a tar product known as pynol, which is made from bone-oil, and is described as "colourless, but becomes brown in air. It has a faint odour like chloroform and a hot burning taste." Iodol itself is a pale yellow crystalline powder, like iodoform in appearance, but without its objectionable taste and odour. It is almost insoluble in water, but is readily soluble in alcohol and ether, less so in glycerine or oils. It is recommended by Erichsen as a substitute for iodoform when the latter is objected to, but it is not so efficacious and is much more expensive. It has the advantage of not being so poisonous as iodoform.

Sozoiodol is another attempt to find a substitute for iodoform. This antiseptic is closely related to the phenols, being a combination of phenol and iodine. It is very slightly soluble in water, but readily in alcohol and glycerine. It is said that iodine is liberated from this substance when exposed to light, which naturally renders it a very unstable product and lessens its usefulness.

Iodoformogen belongs to that recent class of antiseptics where an active antiseptic is combined with a proteid. In this particular instance the combination consists of iodoform and albumen. It is a fine bright yellow, practically odourless powder, which does not tend to roll into balls. It is insoluble in water, but has proved a very efficient and useful dusting powder for wounds, ulcers, &c. Its drawback is that in some cases it has produced eczema.

Antinonnin is the trade name for a patent disinfectant, formed by adding potash salts to a derivation of the higher phenols or cresols. It is a yellowish-coloured paste, which dissolves readily in water, and it is inodorous and non-volatile. It is very poisonous, and has also the disadvantage of staining everything a bright yellow colour. It is, however, a very effectual disinfectant.

### Training in the Provinces.

(*Continued from page* 141.)

### BIRMINGHAM GENERAL HOSPITAL (340 Beds).

### Terms of Training.

Probationers at the Birmingham General Hospital are received for training between the ages of 23 and 35, and are engaged in the first instance for one month on trial. If accepted at the end of that time an agreement to remain in the service of the hospital for three years is signed, for one year of probation, receiving no salary, and for two additional years at a salary of from £16 to £18 per annum. Indoor uniform, three dresses, three caps and six aprons, is provided each year by the hospital, laundry expenses being also defrayed by the hospital. Lectures are given to the nurses by members of the medical staff and the matron, and all nurses are required to present themselves for examination before receiving a certificate on the completion of their training. Salaries for nurses on the permanent staff range from £24 to £28 per annum. Sisters receive from £30 to £36 per annum. Sisters are selected from amongst the best of the staff nurses, a three years' course of training being essential, and generally also some experience as a staff nurse. Staff nurses and probationers take night and day duty in alternate periods of three months.

### Hours On and Off Duty.

Nurses and probationers are on duty in the wards between 7 a.m. and 9 p.m., with time for meals and from one and a half to two and a half hours daily for recreation, the latter being taken alternately from 2 to 4.30 p.m., and from 6.30 to 9 p.m, for staff nurses, 7 to 9 p.m. for assistant nurses, and 7.30 to 9 for probationers in their first year. On alternate Sundays the latter are off duty from 2 to 5 p.m., and from 4 to 10 p.m.; assistant and staff nurses from 10.30 a.m. to 12.15 p.m., and 4 to 10.30 p.m. Probationers have a half-day's leave once a month, assistant nurses a whole day. Staff nurses have leave once a month, from 6.30 p.m. one day to 10 p.m. the next. Sisters are on duty from 8.30 a.m. to 9 p.m., with leave on alternate days from 2 p.m. to 5.30 p.m., and from 5.30 to 8.30 p.m.; on alternate Sundays from 10 a.m. to 1 p.m., and 4 to 10.30 p.m.; and once a month from 2 p.m. on Saturday to 9 a.m. on Monday. Night nurses' hours on duty are from 8.45 p.m. to 8.45 a.m.; they take their recreation from 9.30 a.m. to 12.30 p.m., and on alternate Sundays from 9.30 a.m. to 1 p.m., and 5 to 8 p.m.

### Meals.

All day nurses' meals are served in the nurses' dining room, and no allowances of tea, sugar, &c., are given out to the nurses in weekly stores. The hours for the nurses' meals are as follows: Breakfast, 6.30 a.m.; lunch, 9.30 a.m.; dinner, 12.30 and 1.30 p.m.; tea, 4.30 to 5.30 p.m.; supper, 9 p.m. The sisters' breakfast is at 8 a.m.

---

### QUEEN'S HOSPITAL, BIRMINGHAM (132 Beds).

### Terms of Training.

Applicants for training at the Queen's Hospital, Birmingham, must be between 23 and 33 years of age. A three months' trial is the rule, at the satisfactory conclusion of which accepted candidates are required to sign an agreement binding them to remain in the service of the hospital for four years, two as probationers in the wards, and for the last two either on the external (or private) nursing staff, or as staff nurse in the hospital, at the option of the committee. Probationers receive no salary during their first year; during the second they are paid £15, which is raised to £20 for the third year, and in the fourth to £22 in the hospital or to £25 in the external nursing department. If a candidate gives satisfaction during her first month, at its expiration she is provided with indoor uniform, laundry expenses and indoor uniform being found by the hospital for all the staff, and outdoor uniform in addition for those on the private staff. Salaries for permanent staff nurses vary from £20 to £24, and for sisters from £24 to £30. Nurses on the external staff receive from £20 to £30 per annum and 10 per cent. of the profits on their earnings. Three months' salary is granted to nurses in cases of serious illness. Promotion depends upon merit and capability, no sister being appointed to the charge of a ward with less than three years' training. Lectures are given to the nurses by the matron and by members of the medical staff, and the certificate is granted at the termination of the four years' engagement. Night duty is taken for six months at a time, the second year being divided as nearly as possible into day and night duty.

### Hours On and Off Duty.

Day nurses and probationers are on duty in the wards between 7 a.m. and 9 p.m., two hours off duty being allowed daily from 2 to 4, 5 to 7, or 7 to 9 p.m., with half an hour each for lunch, dinner, and tea. Probationers are also off duty from 3 to 9.30 p m. on one afternoon in the week, nurses and sisters from 3 to 10 p.m. Every other Sunday probationers have leave from 4 to 9.30 p.m., sisters and nurses from 4 to 10 p.m. Sisters are on duty between 7.45 a.m. and 9 p.m., with two hours for recreation daily, and other times as given above. Night nurses are on duty from 9 p.m. to 9 a.m., being daily free from 10 a.m. to 12.30 p.m., and on Wednesdays and Sundays having passes from 4.30 to 8.20 or 8.30 p.m. Nurses on night duty are expected to remain at least seven hours in bed, except on those two days, when they are permitted to reduce the time to six hours. Annual leave is for sisters 19 days and for nurses 16 days.

### Meals.

All meals for day nurses are now served in the nurses' dining-room, the only "stores" given out being for those on night duty for their midnight meal. The hours for day nurses' meals are: Breakfast 6.30 a.m.; lunch, 8.30 to 9 a.m.; dinner, 12.30; tea, 4 p.m.; supper, 9.5 p.m., half an hour being allowed for each meal. Sisters' breakfast is at 7.15 a.m.; lunch is served from 8.30 to 9 a.m.; dinner at 1 30; tea at 4.30; and supper at 9.5. The night nurses breakfast at 8.30 p.m., and their dinner on week-days is at 9.15 a m, on Sundays at 9 a.m.

---

### BRISTOL HOSPITAL FOR SICK CHILDREN (102 Beds).

### Terms of Training.

Probationers are received for two years' training at the Bristol Hospital for Sick Children, paying a premium of £10 and receiving no salary during that period. Laundry and part indoor uniform, consisting of three print dresses, four caps, and four aprons, are provided by the hospital. Out-door uniform according to the hospital pattern must be bought by the probationer. Candidates should be between the ages of 20 and 28 years. Lectures are given by members of the visiting staff on anatomy and physiology, and by the matron on medical and surgical nursing, &c., to the nurses. Nurses who have satisfactorily completed their two years' training may be promoted to staff work for a third year at a salary of £18, but, as there is one permanent staff nurse, only two vacancies occur in the year. There are two permanent night staff nurses and a night sister, the probationers taking three months' night duty at a time as required. There is a convalescent home attached to the hospital, and also an annexe for fever cases, probationers being expected to take duty at either if required. A private nursing staff is also in existence, to which nurses may be promoted after finishing their training. Staff nurses' salaries range from £18 to £28 per annum; sisters' from £35 to £40.

Hours On and Off Duty.

Nurses are on duty in the wards between 7 a.m. and 8 p.m., with time for meals and two hours daily for recreation to be deducted therefrom. On Sundays leave of absence is given alternately from 10 a.m. to 1 p.m. and from 6 to 9.30 p.m. Sisters go on duty in the morning half an hour later than the probationers, and are off duty on alternate days from 2 to 4 p m., and from 6.30 to 10 p.m. A day off, from 10 a.m. to 10 p.m., is given once a month to probationers, and a complete day to sisters and staff nurses. Yearly leave is for probationers a fortnight; for sisters one month; and staff nurses three weeks.

Meals.

All meals for day and night nurses are served in the nurses' dining-rooms, and no allowances of tea, butter, &c., are given into the keeping of the nurses. The hours for meals are as follows: Breakfast, 6.30 a.m.; lunch is on the table from 9 to 10 a.m.; dinner is at 1 and 1.35 p.m.; tea between 5 and 5.45 p.m.; and supper at 8.15 p m. Lunch, tea, and supper for the sisters are served in their own rooms.

### Novelties for Nurses.

THE KNITTED CORSET COMPANY.
(118, Mansfield Road, Nottingham.)

The Knitted Corset Company have recently made some important additions to their excellent assortment of goods which will be found a great comfort to those for whom they are designed. The new corsets for married ladies' wear before and after confinement are designed on a thoroughly practical and original plan. By an ingenious system of lacing the necessary support can be provided where most required without undue pressure elsewhere. There are two or three models that are well worth looking at, and we should advise our readers to send for a catalogue. There are various other delightful articles which maintain the reputation deservedly attained by this enterprising firm, who make a speciality of all kinds of clothing, though woollen garments are their principal line.

CELLULAR CLOTHING.

We can imagine there are few materials more delightful to wear than the cellular cloth recently introduced by Messrs. Oliver Bros., of 417, Oxford Street, and 33, New Bond Street. This charming fabric is manufactured on an entirely new principle, and claims to be warmer in winter and cooler in summer than those to which we have hitherto been accustomed. In the first place, it is porous; and, in the second, it is a non-conductor. Modern science has discovered that air is the best non-conductor of heat, being nearly 100 times more efficacious in this respect than any fibre. It must be remembered that the clothes we wear are not warm in themselves, they only prevent loss of heat from the body in proportion to the amount of air they contain. Cellular cloth is so constructed as to admit the air readily; the two sides are not alike—that which is worn next the skin is open work, while the other side is partially closed by fine threads. It is warm, light, and very durable. We have examined several garments made in cotton, silk, and wool, all woven in this particular style, and were charmed with their appearance and texture. For under garments and bedding it is simply ideal, and we cannot imagine anyone, after having given it a trial, reverting to the old system. It has also the merit of being inexpensive. An excellent quality calico can be had at 7½d. a yard, which is quite good enough for ordinary purposes. Some of the coloured shirtings are perfectly fascinating. One in blue with a narrow white stripe and the same design in pale pink cannot fail to be popular. We also admired a neat white vest with narrow blue line as being very *chic*. Messrs. Oliver also make a specialité of ladies' shirts, the cut and style of which alone should ensure for them a large custom. These are all made to fit the purchaser, and the result is in every way satisfactory. To cyclists and others who indulge in active exercise these garments will be found a veritable godsend, while to invalids and children they are invaluable. We strongly advise their use to all delicate people and travellers to whom warm yet light clothing is an important consideration.

### Appointments.

MATRONS.

The Westminster Hospital Home and Training School for Nurses.—Miss Jessie Southwell has been appointed Matron of the above home for nurses. She was trained at St. Bartholomew's Hospital, acted as night sister and assistant matron at the Chelsea Hospital for Women, and as matron of the Royal Hospital for Children and Women, Bristol.

Aston Manor Nursing Institution, near Birmingham.—Miss C. Coghlan has been appointed Superintendent Nurse of the above institution. She was trained at the Royal Hospital, Portsmouth, and held the post of superintendent at the Hospital for Children, Shanklin, and the Hampstead Nursing Association.

Leavesden Asylum, Watford, Herts.—On July 13th, 1898, Miss Sarah F. Coffey, who was trained at St. Patrick Dun's Hospital, Dublin, was appointed Superintendent Nurse of this asylum. She was afterwards head nurse at a private hospital, Lason Street, Dublin, and has been employed at the Park Hospital, Lewisham.

Royal Cornwall Infirmary, Truro.—Miss E. F. Neve, who was trained at the London Hospital, was appointed Head Nurse and Housekeeper of this infirmary on July 21st, 1898. She has been nurse-matron at Enfield Cottage Hospital.

Cornwall County Asylum, Bodmin.—On May 31st, 1898, Miss M. S. Macarthur was appointed Matron of the asylum. She was trained at Saughton Hall, Gorgie, Edinburgh, where she afterwards held an appointment. She was subsequently charge lady nurse at Wonford House, Exeter.

### "The Hospital" Convalescent Fund.

We have much pleasure in acknowledging contributions towards this most useful fund: 5s. from Nurse Groom, and 5s. from Nurse Edith Boyd. We are glad to tell our readers of another case in which the fund played a most useful part. Nurse H. wrote to us from abroad, where she had gone in the hope of obtaining a complete cure for rheumatism. She had become much worse, and was nearly at the end of her resources. She received a reply that if she would return to England and again visit Droitwich, which had greatly benefited her before, help should be forthcoming. She acted on the advice given, and, after a stay at this watering place, is so far recovered as to be able again to resume a light occupation. Those who have charge of The Hospital Convalescent Fund spare no pains to ascertain the best course to be pursued by suffering nurses who apply for help. A little advice from the experienced is often as helpful as the monetary assistance.

### The Book World for Women and Nurses.

[We invite Correspondence, Criticism, Enquiries, and Notes on Books likely to interest Women and Nurses. Address, Editor, The Hospital (Nurses' Book World), 28 & 29, Southampton Street, Strand, London, W.C.]

A Complete System of Nursing. Written by Medical Men and Nurses. Edited by Honnor Morten. (Sampson Low, Marston, and Company, Fetter Lane, Fleet Street. Pp. 398. Price 7s. 6d.)

Miss Honnor Morten in editing a book extending over so wide a range of subjects has undertaken no easy task, and in saying that she has acquitted herself creditably is only expressing what was to have been expected from her former achievements. The book, though somewhat unequal, is in many parts excellent, and will be found of real assistance to nurses. It does not profess to be a collection of literary essays, but simply an expression of the opinions of certain experts as to the best methods of nursing various classes of disease of which they are qualified to speak from special experience. The chapter on hygiene of the sick-room, for instance, is eminently practical, and if the directions given were more carefully attended to they would largely diminish the tendency that exists towards that condition known as "under health" in those who live in unventilated dwellings. Some of the subjects subsequently dealt with are perhaps unduly compressed, and others a little inclined to be prolix, but this is the inevitable result of papers being contributed by different writers, uniformity of standard being under such circumstances impossible. There are many invaluable hints given on the nursing of infectious fevers, also on ophthalmic nursing, both of which have special points of interest differentiating them from every-day nursing, points which the ordinary nurse has no means of making herself acquainted with. The chapter on massage and electricity also will be found of use, the instructions given being very plain and easily carried out. Several pages are devoted to gynæcological and monthly nursing, which alone will ensure for the book a position as a work of reference. The chapter on "The Nursing of Babies" is admirable, and should be read by all mothers as well as nurses. The handling of a baby is an art in itself, and one too often, alas ! neglected, with possible injury to the child far-reaching in its results. It would be well if every woman who has to deal with children would commit to memory the excellent paragraph on "The Daily Bath," and practice the regulations it recommends. The merits of this handy little volume are considerable; to say it is without fault would, perhaps, be going too far, but the faults are of a nature that can easily be rectified in the next edition without in any way altering the nature and scope of the book, which deserves to be widely used by the class for whom it is intended.

"The Angel Hermit." By A. W. Letts, Author of "Carmina Fidelium," Illustrated by W. F. Southcott, Dedicated by special permission to H.R.H. the Duchess of York, in Memory of her late lamented Mother. (London : Simpkin, Marshall, Hamilton, Kent, and Co., Limited). Price 2s. 6d.

Mr. Letts tells an exceedingly pretty legend in tolerable blank verse. An angel, oppressed by the thought of the sins of mankind, offers to occupy a hermitage in a central part of England, and give his heavenly comfort to such as may come to him for help. The condition on which he is permitted to fill this office is that the sins of everyone whom he soothes shall be a burden to himself; and he is to remain in the hermitage until some mortal comes who is willing to take up the task. It is many years before a knight appears, who has fought in the crusades, but has found the slaying of paynims no consolation to his sin-burdened soul. To him the angel confides the truth about himself, and the knight offers to relieve him and to find his own salvation in the comfort he can give to the pilgrims who may come to the shrine. We have said that Mr. Letts's blank verse is only tolerable ; but it is right to add that some portions of his narrative which are told in rhyme are of a much higher poetical quality. One hymn, which is introduced, is genuinely beautiful. We could wish that the legend had been told in a poetical prose, which we think Mr. Letts could easily write, with the lyric portions of the present poem introduced. In such a shape, we think, the legend would be even more attractive than it is now. But it is worth reading in its present dress. We may add that the proceeds of the publication are to be given to charities.

"Billy," and Other Stories. Henry Clarke. (London : Simpkin, Marshall, Kent, and Co. 1898.)

In the preface to this volume of short stories the author explains that they were written for and published in "The Birmingham Central Literary Magazine," a quarterly periodical issued by the members of the Central Literary Association of Birmingham, an association from which has sprung some of Birmingham's most capable and honourable public men. For this reason alone the book commends itself, but "Billy" and the other sketches here published have merit other than for their actual history. Mr. Henry Clarke shows no inconsiderable a power of writing, and a wide experience of human life. The stories here collected together are of a varied nature, embracing such subjects as "The Heathen Chinee," "Is Marriage a Failure," "A Night on the Deep," "The Faces of Spring Flowers," &c., and we shall look for more good work from Mr. Clarke's pen.

### MAGAZINES OF THE MONTH.

The English Illustrated Magazine for July has excellent contents within its attractive cover. "A Man's Chance of Life," by Holt Schooling, with diagrams, is interesting, and will appeal to us all. A humorously written story is "The Hamilton Honeymoon," by Charles D. Leslie. "The Writing Master of Gore" is well illustrated, and gives an epitomised history of the copy-book. The first book of which description, according to our authority, was "a booke containing divers sorts of hands," which was "imprinted at London by Thomas Vantroueller, dwelling in Black Friers 1571." There is also an article on the great Napoleon dealing with the invasion of Russia, which forms most interesting reading.

Cassell's Magazine for July has a charming coloured cover, and the contents sustains the excellent standard which "Cassell's Magazine" always attains. Here in the current number there is a pretty little story called "The Mayor's Japanese Wife," by Douglas Sladen, and Tighe Hopkins contributes a complete story, "The Footprint of Princess Trubelskoi." Other articles are, "American Women in War Time"; "Houseboat Isis: A Romance of Henley Regatta"; "Public Receptions at the White House," by Elizabeth L. Bankee ; and "Big Guns in Action," by an Artillery Officer.

The Harmsworth Magazine for July, in its first appearance has fully realised all expectations Here we find over twenty articles and 200 illustrations, which means a picture to every page. In the preface Messrs. Harmsworth say: "We are enabled to issue a threepenny magazine containing more expensive literary matter, more numerous pictures, and more pages than the sixpenny magazines, because this magazine is only a small incident in an organisation controlling four daily journals and nearly three weekly periodicals." Even with this explanation, it is difficult to understand how so admirable a magazine can be produced for the small sum asked for it.

### Everybody's Opinion.

[Correspondence on all subjects is invited, but we cannot in any way be responsible for the opinions expressed by our correspondents. No communication can be entertained if the name and address of the correspondent is not given, as a guarantee of good faith but not necessarily for publication, or unless one side of the paper only is written on.]

### MATRONS' COUNCIL MEETING.

"H. E. B." writes: I also, as well as "A Constant Reader," do not understand why nurses who have been trained in small hospitals should not have as good a standing as those trained in large ones. After completing six months' training in a Q.V.T. affiliated home, the superintendent, who knew every detail of my work, sent my name to the Queen's Inspector as a "competent and trustworthy" nurse for enrolment as a Queen's nurse. The answer came back that I could not be accepted, because the hospital in which I was trained had only contained seventeen beds (I had had nine months' experience in a hospital of fifty-six beds as well). The Inspector kindly offered to send me into a hospital for twelve months' further training. Fancy an enthusiastic district nurse who knows she is competent returning to hospital life! I am now in a non-affiliated home.

"THE MATRON OF A SMALL HOSPITAL" writes: I was very glad to read the letter from "Sister Mary" and "Old Re——l" in connection with training in provincial hospitals. I am sure the training in some of the small hospitals is quite as good as the training in the hospitals of our "great City." It is a pity that the time spent in a smaller hospital does not count for more. I am sure some of our best nurses are trained in provincial institutions. What is the use of a theoretical nurse if she is not practical and does not possess the qualifications so essential? I spent my first year is a small hospital, and I have worked since in two of our largest hospitals. I must say I saw more of the actual surgery and had more dressings to do during my first year than after it, for in the larger hospitals the students do most of the dressings, so that only the clearing away is left to the nurses. Let me give a little incident of a London-trained nurse which occurred in my small experience. She was sent to take night duty at a private case that I was nursing, and had had three years' training in one of the London hospitals. The medical man told her to pass a rectal tube. The trained London nurse had no idea what a rectal tube was or how to use it. I do not wish to infer that this is the case with all London trained nurses.

### NURSES FOR BOARD SCHOOLS.

MISS MARY WARDELL writes: Your article in THE HOSPITAL for July 23rd, respecting nurses for Board Schools, induces me to venture a suggestion. Why need thoroughly trained certificated nurses be employed? If thoroughly-capable women of the type of domestic nurse were employed, surely they would suffice to discover vermin, tie up a cut finger, &c. In fact, I think that class attend more thoroughly to smaller details needing motherly care than a nurse preferring heroic cases calling out special energy to pull a patient through a serious illness. There would also be less temptation to act the doctor. Another remark on a different subject I venture to make. In reference to the anti-phosphorus movement, I see matches non-phosphorus able to be struck on anything are being advertised. Would not these matches greatly increase the risk of household fires?

*** We urged that nurses should not encroach upon the province of the qualified practitioner, but that the work required is more than could be undertaken by a woman who could merely wrap up a cut finger is equally apparent. —ED. *T. H.*

### The Meath Home of Comfort for Epileptics.

THE Meath Home of Comfort for Epileptic Women and Girls, Westbrook, Godalming, Surrey, is one of the three establishments in England for this class of patient. Patients are admitted from the age of two to thirty-five; they receive every care, and are trained in industries that not only exercise a beneficial effect on their health and happiness, but by the sale of the products add to the funds of the home. The Meath Home was founded five years ago after long thought by Lady Meath, who, as Lady Brabazon, established the Homes of Comfort for working girls at Reigate. The roomy old-fashioned residence situated in charming and picturesque grounds was an excellent bargain, for the railway company had requisitioned a part of the garden for the station at Godalming, and the house in consequence had been to let for seven years. The committee have incurred considerable expense in fitting it up with the necessary appliances for the health and comfort of the inmates. The walls are covered with cheerful papers, the baths and lavatories are modern, and the furniture good, suitable, and sufficient.

Two classes of patients are admitted; gentle and working women. The ladies' rooms are divided into cubicles by white curtains. A marble-topped washstand and a dressing chest replace the locker of the second grade, but both classes of accommodation are equally comfortable.

We regret to learn that for the first time the reserve fund of £500 has been considerably encroached on, and that the committee in consequence have issued an earnest appeal for help. Anyone glancing through the accounts will note, however, that repairs to old buildings amount to £174, and connecting the drainage of the home with the main system of the borough has cost £162. Both outlays were essential and justify the appeal, but it is somewhat misleading to find such items entered in the ordinary expenditure. These and some other entries of smaller amounts ought to be counted as extraordinary.

On Monday, 25th, the anniversary festival was held at the home. The guests assembled in the church, and after evensong the Rev. C. J. Ridgeway, of Christ Church, Lancaster Gate, preached for the charity, and a collection was taken. The company then assembled on the lawn of the home, and the girls sang a cantata very creditably. They were dressed in white or coloured muslin dresses, cut Greek fashion, and carried boughs in their hands. They represented a band of fairies, and entered into the spirit of the piece with great zest. Mrs. Frank Mellersh and her co-adjutors have reason to be well satisfied with the result. Tea was served after the performance in the garden, and a band played frequently. The home was open for inspection, and the girls were busy with their various employments, seated out-of-doors. Baskets, clothing, and articles of needlework were on sale, and the proceedings were satisfactory in every way.

The proximity to the railway station is a great convenience, as patients can be brought to and from the home without passing through the town.

### Minor Appointments.

THE GENERAL INFIRMARY, MACCLESFIELD.—Miss M. Carruthers has been appointed Sister of the children's ward of the above hospital. She was trained at the Cumberland Infirmary, Carlisle, for three years, and worked afterwards as charge nurse at the same institution. She has since held the post of charge nurse at the Chesterfield and North Derbyshire Hospital.

ST. JOHN WORKHOUSE INFIRMARY, HAMPSTEAD.—Miss Helen Goldsmith Grant has been appointed Night Sister at the above infirmary. She was trained at Crumpsall Infirmary Manchester, for three years, and has since held the post of superintendent nurse at the Holborn Workhouse Infirmary, Mitcham.

### For Reading to the Sick.

### PROSPERITY AND ADVERSITY.

**Verses.**

Now—the spirit, conflict-riven,
Wounded heart, unequal strife!
Afterward—the triumph given
And the Victor's crown of life!

Now, the training, strange and lowly,
Unexplained and tedious now!
Afterward—the service holy,
And the Master's "Enter thou."

—*F. R. Havergal.*

Who would dare the choice, neither or both to know,
The finest quiver of joy, or the agony thrill of woe?
Never the exquisite pain, then never the exquisite bliss;
For the heart that is dull to that can never be strong to this.

—*F. R. H.*

Thou hast done well to kneel and say,
"Since He Who gave can take away,
And bid me suffer, I obey!"
And also well to tell thy heart
That good lies in the bitterest part,
And thou wilt profit by her smart. . . .
Nor with thy share of work be vexed;
Though incomplete and ev'n perplext,
It fits exactly to the nest.
What seems so dark to thy dim sight
May be a shadow, seen aright,
Making some brightness doubly bright.
The flash that struck thy tree—no more
To shelter thee—lets Heaven's blue floor
Shine where it never shone before!

—*A. Procter.*

We wish that men by men despised,
And such as lift their foreheads over-prized,
Should sometimes think . . .
What recompense is kept in store or left
For all that seems neglected or bereft;
With what nice care equivalents are given,
How just, how beautiful, the Hand of Heaven.

—*Wordsworth.*

**Reading.**

God has His own way of training our souls, and we may take the particular circumstances of our life as making the particular form of training that is necessary for our soul. Sometimes we are tempted to think we could be so good if our circumstances were only different; if we could only get away from the turmoil and noise and worry of life it would be so easy to be good, but when it is manifestly God's will that we should live in the middle of worries and troubles and temptations, we must take it as a mark of His will that those are the means by which He wishes to humble us, and make us like Himself; and if we were in a cowardly manner to run away from them we should only run into other and more serious temptations, and certainly become worse instead of better.

God has different ways of treating different souls. Some people seem to have everything easy; they have a great devotion, and are able to express it easily. Others are cold and hard and get very little happiness out of religion. . . .

The fact that God calls many more people to Him through tribulation and spiritual trials than He does by giving either the good things of this world or the next, certainly points to the conclusion that it is easier to serve God faithfully in trouble than it is in happiness, and there is no doubt that it is often the fact that people who seek God earnestly in trouble, forget all about religion when their troubles are removed and they become prosperous.

### Presentations.

MRS. MACDOUGALL, who has been matron of Morningside Asylum for 30 years, was, at a staff dance, presented with a silver coffee-pot from her nurses, and kitchen and laundry staff. Dr. Clouston, in making the presentation, bore testimony to Mrs. Macdougall's great interest in and motherly care of the patients and staff, and he congratulated her upon retiring in good health after so long an official life.

### Notes and Queries.

**The contents of the Editor's Letter-box have now reached such unwieldy proportions that it has become necessary to establish a hard and fast rule regarding Answers to Correspondents. In future, all questions requiring replies will continue to be answered in this column without any fee. If an answer is required by letter, a fee of half-a-crown must be enclosed with the note containing the enquiry. We are always pleased to help our numerous correspondents to the fullest extent, and we can trust them to sympathise in the overwhelming amount of writing which makes the new rules a necessity.**

**Every communication must be accompanied by the writer's name and address, otherwise it will receive no attention.**

*"Ameryl" Soap.*

(165) Can you tell me if the "Ameryl" soap sold for reducing fat is efficacious and injurious?—*Silla.*

We cannot answer the first question, but we do not think that it is injurious.

*Raynaud's Disease.*

(166) Can you tell me where I can read up about Raynaud's diseases, where the hands and feet turn purple and black when cold). I have a case for massage and should like to know all about it.—*Silla.*

Most manuals of the practice of medicine describe the phenomena of Raynaud's disease, as, for example, Osler's medicine. Its origin is very obscure. The cause of the visible mischief is no doubt a local and symmetrical spasm of the small blood vessels; and, again, there is hardly a doubt that the stimulus in response to which the muscular fibres in the blood vessels contract reaches them by way of the nervous system. But where it comes from, whether it is a toxic affair or the result of central disease, or even as some would say a "reflex" spasm, is not so easily decided. What is certain is that these cases sometimes react very curiously to friction and to changes of temperature, and it is therefore important that the nurse should very carefully follow the directions given her, and also that she should very carefully observe what changes follow her manipulations, not merely immediately but after an hour or more. One hint we would give. A nurse should carefully render her hands aseptic before using massage in such a case, for the vitality of the skin is at a low ebb, and the slightest abrasion, if infected with septic organisms, may readily lead to ulceration and gangrene.

*Probation in Children's Hospital*

(167) I am desirous of obtaining a place as probationer in a children's hospital, preferably in Wales or in the country. I would require a salary. My age is 23. I am well educated, but have no experience of nursing.—*Pauline.*

Rhyl Royal Alexandra Hospital for Children. Apply Hon. Lady Superintendent. A personal interview, a premium of £5, and a month's trial necessary. Salary first year £12, uniform, and laundry. A three-year certificate granted.

*Eczema.*

(168) I have slight eczema on both hands. Will it disqualify me for the nursing profession?—*H. H. M.*

Certainly. The slightest break in the skin of a nurse's hands is a source of danger.

*Holiday Homes.*

(169) Could the Editor kindly give addresses of nurses' holiday homes in Bournemouth and Weymouth?—*S. M. D.*

Fairleigh, Drummond Road, Boscombe, Bournemouth, 18s. weekly; Winterslow House, Weymouth, from £1 1s. weekly. If "S. M. D.'s" choice is not limited to Bournemouth and Weymouth, she may find other homes at more moderate charges in "Homes and Hospitals for the Benefit of Gentlewomen," price 1s., from the Army and Navy Stores, Victoria Street, Westminster.

*Nurse for Johannesburg.*

(170) E. R. would be very glad if the editor could give her the address of the nursing institution in Johannesburg. She is anxious to go to Africa, and would like to know where they require nurses.

Sister Adele is the name of the lady superintendent of the Johannesburg hospital. She would possibly give E. R. the information she wants.

*Maternity Pupil.*

(171) Please tell me at what age hospitals take probationers. After general training I wish to be trained in a lying-in hospital. Is a good education necessary, as I have only had a Board school education?—*A. B.*

Few hospitals care for probationers under 23. Lying-in hospitals train maternity pupils from 20 to 45 years of age. A Board school education is good enough if the candidate is otherwise suitable.

*A Leper Missionary.*

(172) Can you give me Miss Kate Marsden's address? She has joined some mission to lepers. She lectured some time ago on Churches, but I don't know if she is the lady of Siberian fame.—*E. M. M.*

We have not her address. She has written a book upon the lepers of Siberia.

